# Single‐channel EEG classification of sleep stages based on REM microstructure

**DOI:** 10.1049/htl2.12007

**Published:** 2021-04-20

**Authors:** Irene Rechichi, Maurizio Zibetti, Luigi Borzì, Gabriella Olmo, Leonardo Lopiano

**Affiliations:** ^1^ Department of Control and Computer Engineering Politecnico di Torino Torino Italy; ^2^ Department of Neuroscience “Rita Levi Montalcini” Università degli Studi di Torino Torino Italy

## Abstract

Rapid‐eye movement (REM) sleep, or paradoxical sleep, accounts for 20–25% of total night‐time sleep in healthy adults and may be related, in pathological cases, to parasomnias. A large percentage of Parkinson's disease patients suffer from sleep disorders, including REM sleep behaviour disorder and hypokinesia; monitoring their sleep cycle and related activities would help to improve their quality of life. There is a need to accurately classify REM and the other stages of sleep in order to properly identify and monitor parasomnias. This study proposes a method for the identification of REM sleep from raw single‐channel electroencephalogram data, employing novel features based on REM microstructures. Sleep stage classification was performed by means of random forest (RF) classifier, K‐nearest neighbour (K‐NN) classifier and random Under sampling boosted trees (RUSBoost); the classifiers were trained using a set of published and novel features. REM detection accuracy ranges from 89% to 92.7%, and the classifiers achieved a F‐1 score (REM class) of about 0.83 (RF), 0.80 (K‐NN), and 0.70 (RUSBoost). These methods provide encouraging outcomes in automatic sleep scoring and REM detection based on raw single‐channel electroencephalogram, assessing the feasibility of a home sleep monitoring device with fewer channels.

## 1 INTRODUCTION

Human sleep, according to the Rechtschaffen and Kales (R&K) scoring criteria [1], is divided in stages *N*1 to *N*4 (from light to deep) and stage R (rapid eye movement – REM [2]). This latter exhibits mixed frequency, low voltage, skeletal muscle atonia and bursts of rapid eye movements [3]. It accounts for 20–25% of total night‐time sleep in healthy subjects, and plays a fundamental role in the processing and consolidation of memories [4] and in the regulation of emotional states [5]. On the other hand, the REM behaviour disorder (RBD) [6] is a parasomnia featuring loss of physiological muscle atonia during REM sleep and abnormal sleep behaviour, manifested by dreams enactment and increased violence [7]. RBD is related to poor quality of life (QoL) and psychological disorders such as anxiety [8]. Evidence shows that RBD is a precursor to Parkinson's disease (PD) and other neurodegenerative diseases [9], and about 40% of PD patients complain of RBD and nocturnal hypokinesia, [10]. More in general, sleep disorders are increasing in the aging population worldwide, and simple sleep monitoring systems would help improving their QoL. The gold standard to diagnose and monitor sleep disorders is polysomnography (PSG), a collection of recordings that include electroencephalogram (EEG), electrooculogram (EOG), electromyogram (EMG), electrocardiogram, as well as pulse oximetry and photoplethysmography. However, PSG is costly, impractical and inconvenient for patients, because of the high number of electrodes employed and the non‐familiar environment. Actually, the test should be performed over two nights, as the first one is affected by the ``first night effect${}^{\prime \prime }$, *that is* very low sleep quality. Moreover, PSG recordings are scored manually by a sleep expert, and the rating process is subjective and time‐consuming (the medical score is often available after many working days). Different studies in literature aim at providing automatic multi‐stage sleep classification using several PSG signals [11, 12, 13], with results comparable to expert annotations, but considerable complexity. Other studies address only EEG channels to perform multi‐stage or stage‐specific classification, achieving good performance [14, 15]. Some of these works adopt a feature‐based approach [16, 17], while more recent ones address deep learning (DL) [18, 19] and attention mechanisms [20] on data from one or two EEG channels [21], with good performance. Hence, even though PSG and visual scoring of sleep epochs remain the clinical gold standard, automatic sleep scoring represents a promising approach for sleep disorder follow‐up and longitudinal studies. The main contributions of this work are the implementation of algorithms for automatic sleep scoring (with a focus on REM sleep identification) from a single‐channel raw EEG, and the definition of novel features based on REM sleep micro‐structures. It represents a feasibility test for sleep monitoring based on a single electrode, possibly to be performed at home, thus alleviating the inconvenience for the subject and the sleep expert.

## 2 DATA

The data employed in this work belong to the DREAMS Subjects Database [22], available online. It is a collection of PSG recordings from 20 healthy individuals (four males) with neither underlying neurological pathology nor sleep disorders. At the time of recording they were taking no medication. Most subjects are aged 18–25, but 25% of the participants belong to the 45+ class (age 33.5 ± 14 years). The mean recording time is 8h 30m. All PSG recordings were annotated by an expert, according to both the R&K and the American Academy of Sleep Medicine (AASM) criteria [23]. The AASM annotation, used in this paper, scores each 30 s epoch in one out of five stages: awake (AWA), non‐rapid eye movement sleep (arranged in stages *N*1, *N*2, *N*3, from light to deep) and REM. Ten subjects have been included in our study, for which regular sleep cycles can be identified, and at least 50 min REM periods are indeed present. The other PSGs have been discarded due to the irregularity of the sleep cycle or the absence of REM and *N*3 epochs, possibly due to the first‐night effect [24]. According to the AASM criteria, non‐classifiable sleep stages are labelled either 0, −1 or −2. These epochs are discarded too as they are not relevant. The final dataset is made of *N* = 8.382, 30 s epochs. As detailed in Table 1, the dataset is quite imbalanced towards the *N*2 class, a common situation because *N*2 accounts for 45–55% of the total sleep cycle [25]. On the other hand, *N*1 is very little represented, as it represents a transitional stage from AWA to *N*2. The EEG signals have been re‐sampled at 256 Hz. All recordings have been pre‐processed to reduce high‐frequency noise and remove slow drifts. The signals were high‐pass filtered with an IIR Chebyshev type I, order 1, cut‐off frequency 0.5 Hz, and low‐pass filtered with an IIR Chebyshev type I, order 11, cut‐off frequency 40 Hz. In both cases an anti‐causal filter (zero lag) has been used to avoid delay. Since the algorithm is based on raw EEG data, no further processing has been performed (e.g. no artefact removal, no spatial filtering).

## 3 THE MICRO‐STRUCTURE OF REM SLEEP

**FIGURE 1:**
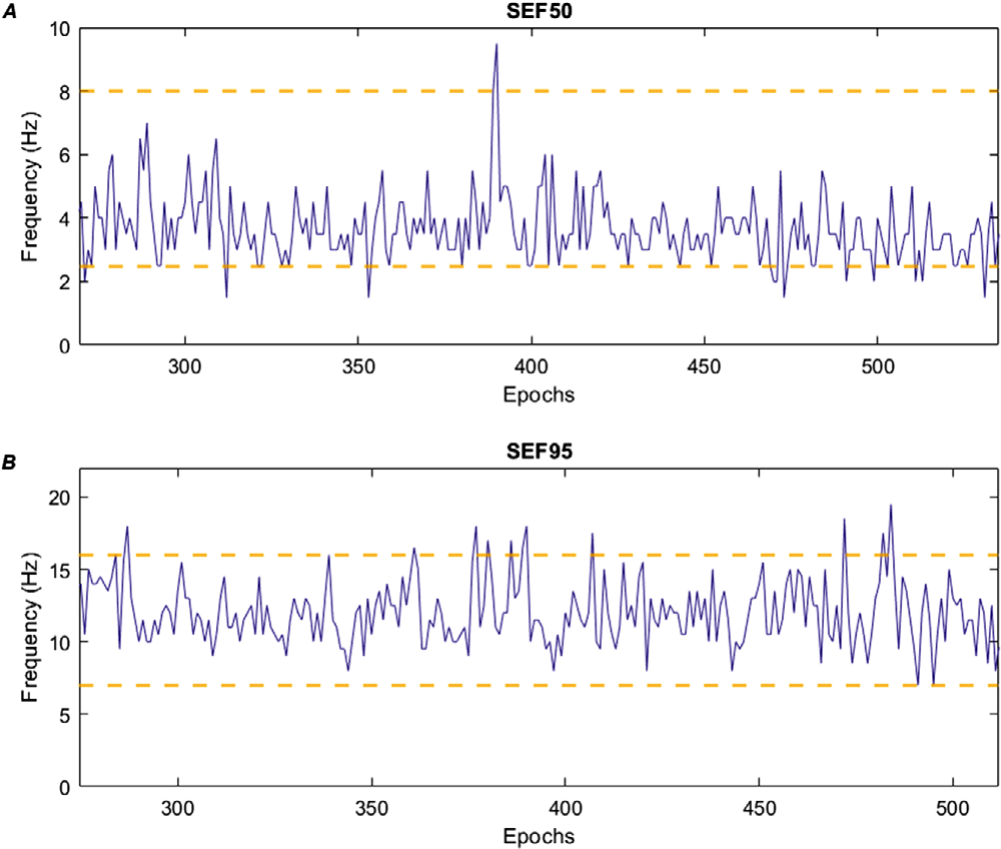
Sample (A) SEF50 and (B) SEF95 as functions of the epoch number

**FIGURE 2:**
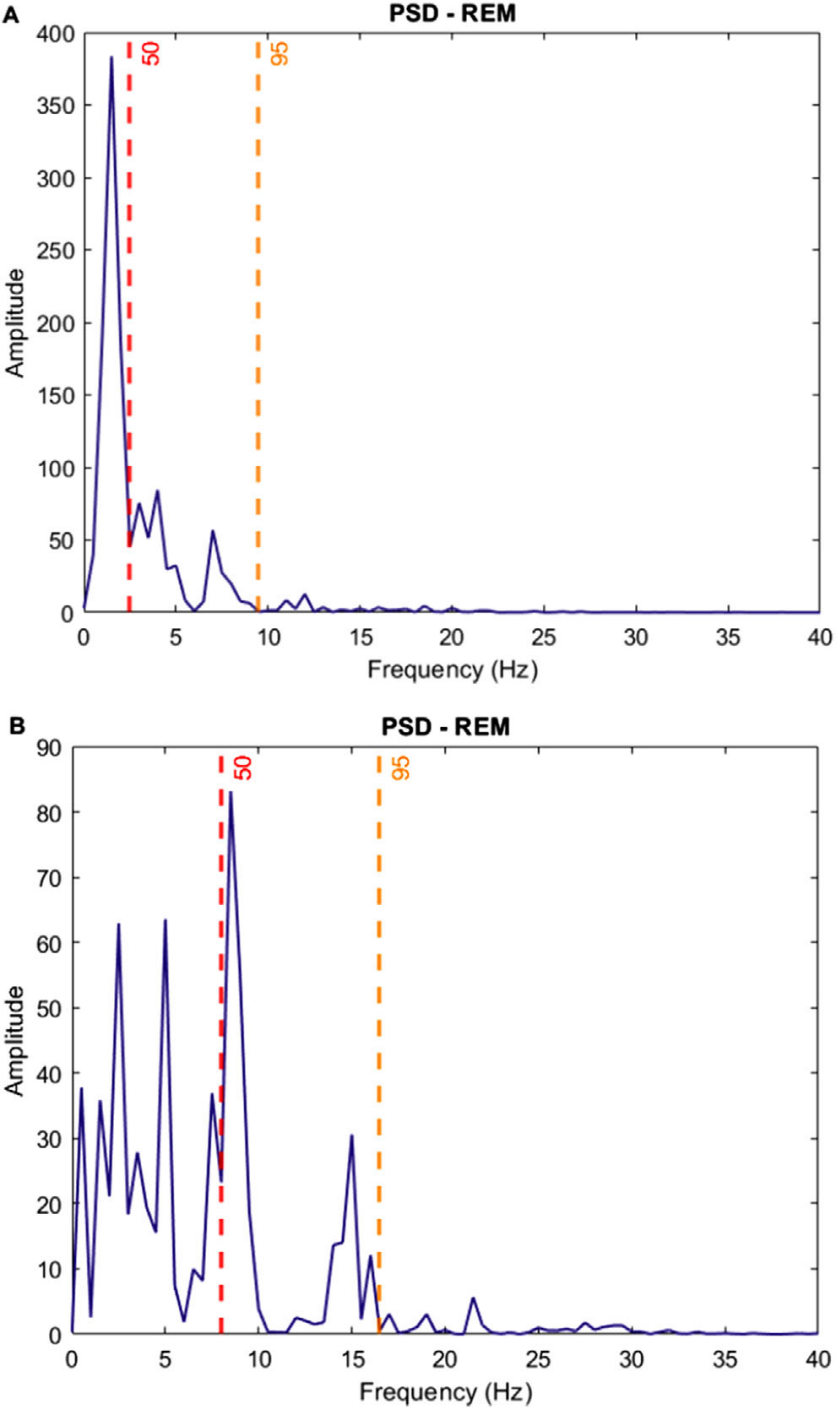
Power spectral densities of typical (A) FREM and (B) TREM micro‐states. Median frequency (in red) and spectral edge frequency at 95% (orange) are shown

Even though it is commonly treated as a homogeneous state, the first evidence of the presence of two micro‐states in paradoxical sleep dates back to the 1960s [26]. Such micro‐states, denoted as phasic and tonic periods, represent markedly different brain states as regards cortical activity and information processing [3], and alternate during REM sleep. The tonic stage (TREM) is the longest and most quiescent one. It consists of segments with no significant ocular movements (EOG depolarizations lower than 25 μV in a 4 s range) and features muscle atonia. On the other hand, the phasic stage (FREM) is characterized by bursts of rapid‐eye movements (at least two consecutive depolarizations in a 4 s range), sawtooth waves, and irregular cardiac and respiratory activity. In automatic classification based on EEG signals only, REM sleep can be mistaken for AWA or *N*1 as it exhibits low‐amplitude and mixed frequency components. Hence, given that TREM and FREM show very recognizable characteristics, a key idea of this paper is to define features typical of either micro‐state, and use them, in addition to others, to feed the classification algorithms. In this way, the classification performance should improve, especially as regards REM stage. In more detail, it is known that the micro‐states differ in terms of power spectral density (PSD): TREM (FREM) exhibits increased power in the alpha and beta (delta and theta) frequency ranges respectively [27]. Hence, we define two frequency bands significant for extracting features specific of either REM micro‐structure: FREM and TREM bands. First of all, we estimate the PSD for each REM epoch. Then, we evaluate the median frequency (SEF50) and the spectral edge frequency at 95% (SEF95), that is the frequency below which 95% of total power lies. Finally, the FREM (TREM) frequency bounds are obtained by averaging SEF50 and SEF95 values belonging to the 25th and 75th percentiles of the related distributions, respectively. The obtained FREM and TREM bands turned out to be 2 – 8 Hz and 7 – 16 Hz, respectively. In Figure 1, sample SEF50 and SEF95 are shown as functions of the epoch number; the frequency bounds are reported as dotted lines. A similar approach is described in [28] and used to distinguish REM sleep from AWA and S1. Moreover, in [14] similar results are obtained based on the epoch PSD. As an example, in Figure 2 the power spectrum of two different REM epochs is depicted. The spectrum of Figure 2(A) is skewed towards the lower frequencies, suggesting a FREM behaviour whereas in Figure 2(B) the spectrum is more peaked and centred in the alpha and beta bands, suggesting a TREM micro‐state. The values of SEF50 and SEF95 are reported as dotted lines and reveal a good accordance with the frequency bands defined in the present work.

**TABLE 1:** Number of epochs for each sleep stage

**AWA**	**REM**	*N* **1**	*N* **2**	*N* **3**
951	1347	555	3791	1738

## 4 FEATURE EXTRACTION

In this work, a total of 164 features have been extracted from the dataset and divided in four categories: time, frequency, time‐frequency and non‐linear. A combination of features already proposed in literature and novel ones is employed. Time features have been calculated for each 30 s epoch, whereas features belonging to the other three classes have been evaluated in each 1 s sub‐epoch and then averaged across the 30 s epoch. This approach is widely adopted in sleep stage classification, with typical sub‐epochs of 2, 5 or 10 s [12, 14]. In fact, given that the EEG signal is not stationary, shorter windows guarantee wide‐sense stationarity. Furthermore, the method provides an adequate spectral resolution (1 Hz). A list of the extracted features is described in Table 2, possibly along with the reference of the paper(s) where they have been proposed. Many features are self‐explaining. The discrete wavelet transform (DWT) was applied to the EOG signal in [13] and [31]. In this work it is applied to the EEG signal, and several related numerical and statistical measures are used as features. The Teager–Kaiser energy operator (TKEO) has been calculated for the whole spectrum (0 – 40 Hz) and its numerical and statistical measures adopted as features, whereas in [31] only two power bands were taken into account. All features have been subjected to min‐max scaling, setting the normalized range in [−1,1]. As already discussed, the novel features proposed in this work are based on the REM sleep micro‐structure and encompass the absolute and relative power in FREM and TREM bands, along with the energy density in these frequency bands, the spectral features SEF50, SEF95 and the differential frequency (SEFd), which consists in the difference between SEF95 and SEF50 [14]. As for feature selection, we have evaluated the variance of the extracted features and removed those with negligible variance; a threshold of 0.2 (heuristically selected) was applied and all the features not meeting this criterion removed. The 87 remaining features have been used to train supervised models, as described in the following section.

**TABLE 2:** Adopted features, along with proper references

**Category**	**Name and description**	**Ref**.
Time	Numerical and statistical measures (mean, standard deviation, skewness, kurtosis, range, max, min)	various
	Hjorth parameters (signal and its derivative)	[29]
	Zero crossing rate	[30]
	25th, 75th, 95th percentile and their differential	various
	Envelope: number of peaks, peak prominence, peak width	^**^
	Coastline (first and second derivative)	[13]
Frequency	Power percentage for each clinically relevant band	various
	SEF50, SEF95, SEFd, absolute power, relative power (TREM)	^*^[14]
	SEF50, SEF95, SEFd, absolute power, relative power (FREM)	^**^
	Entropy and approximate entropy	^**^
	Fast Fourier transform: numerical and statistical measures	various
	Relative power for each clinically relevant band	various
	Energy density in tonic and phasic REM	^**^
Time‐frequency	Short time Fourier transform: magnitude and maximum value of its density (0 – 40 Hz)	^**^
	Discrete wavelet transform coefficients: Daubechies order ouri and Haar filter wavelet	[13]
Non‐Linear	Teager‐Kaiser energy operator: numerical and statistical measures	^*^[31]

^*^ adapted from the indicated study

^**^ novel features proposed in this study

## 5 AUTOMATIC SLEEP STAGE CLASSIFICATION

We applied a non‐parametric classification method (K‐NN) and two ensemble learning methods (RF, boosted trees), described in the following, along with the main relevant parameters.

K‐NN classifies observations based on their similarity to a given metric. It assigns a weight to each observation, depending on its distance to the other points in the dataset. Then, it selects the *K*‐top observations, that is closest to the example, and chooses the most recurrent label. In this work, after heuristic optimization, the classification parameters are set as follows:

- Number of neighbours *K* = 10;
- Distance measure: Euclidean distance.

RF is an ensemble learning classification method, and consists of a high number of decision trees. Each tree is provided with a random subset of the available observations; each node of the tree uses a randomly selected subset of the provided features – thus reducing the risk for overfitting. The chosen parameters are:

- Number of learners: 30;
- Maximum number of nodes: 0.2 · NF, with NF being the number of features used to train the model.

RUSBoost is a particular division of boosted trees, in the form of random‐under‐sampling. This class of random forests has proved to perform well when learning from imbalanced datasets [32], a common situation in sleep stage classification patterns. The algorithm takes *N* as the basic unit for sampling. *N* is picked as the number of observations of the least represented class – in our database, this corresponds to *N*1 class. Classes with more observations are under‐sampled to *N*, in order to obtain a balanced dataset. The number of learners is again set at 30.

## 6 RESULTS OF 5‐STAGE CLASSIFICATION

We have employed the described algorithms to address the 5‐stage sleep classification problem (*N*1–*N*3, AWA, REM) using data from healthy subjects. Performance is evaluated in terms of sensitivity, specificity, accuracy, precision, and F1‐score. Table 3 reports the micro‐averaged performance of the RF classifier; micro‐averaging was chosen in order to take dataset imbalance into proper account, and *k*‐fold cross validation (*k* = 10) is addressed. The performance is generally satisfactory, with the exception of *N*1 stage, which exhibits low sensitivity (hence, precision and F1‐score). This behaviour is shared by all the tested algorithms. As already discussed, this is due to the peculiarity of *N*1, which is poorly recognizable and can be better described as a transition between AWA and *N*2 than an independent stage; this makes the opportunity of its inclusion in the classification task questionable. The impaired performance on *N*1 is also related to the dataset imbalance, as this class is very little represented (about 6% of the total sleep epochs), and under‐sampling is not recommended, as it would waste most information. In any case, it can be appreciated that RF achieves an average accuracy of 83.3%, evaluated as in Equation (1) with TP, TN, FP, FN being true positives (negatives) and false positives (negatives) respectively:

(1) $$accuracy=\frac{TP+TN}{TP+TN+FN+FP}$$

**FIGURE 3:**
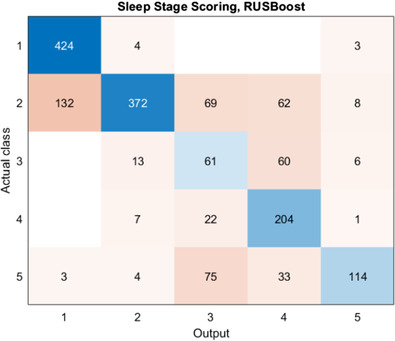
Confusion matrix yielded by the RUSBoost classifier. The class labels (1–5) represent, in order, *N*3, *N*2, *N*1, REM, AWA

**FIGURE 4:**
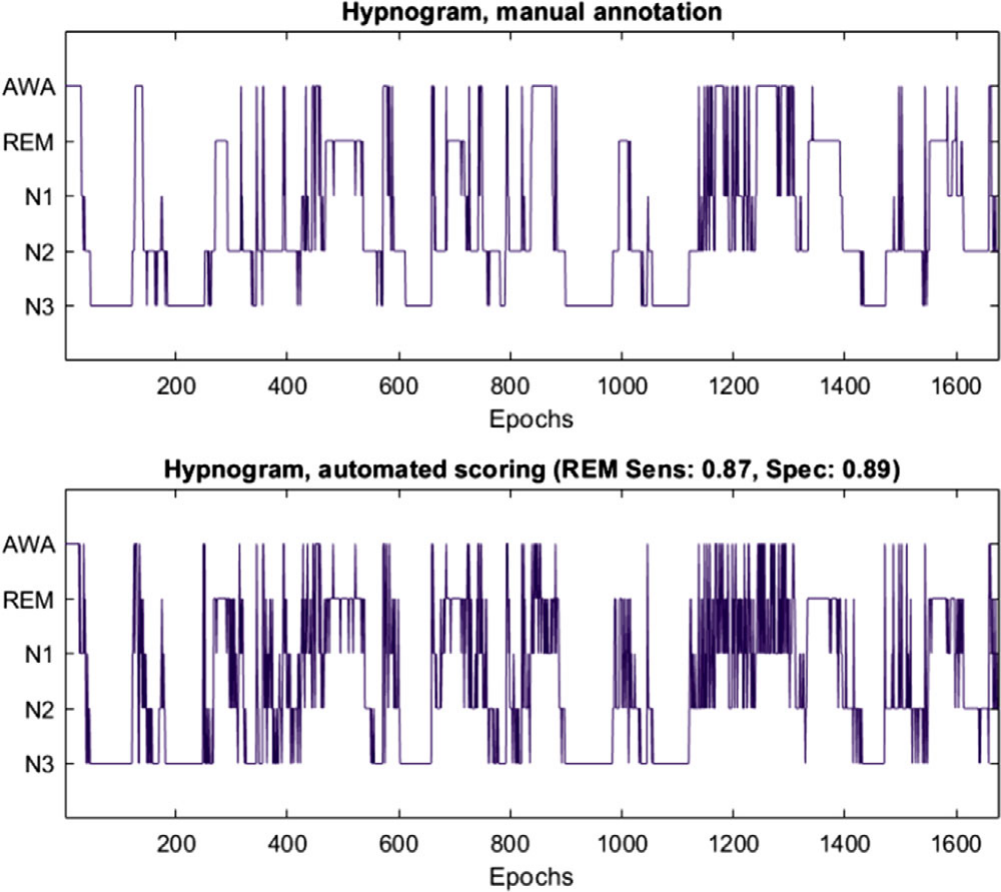
An example of comparison between hypnogram manual annotation and RUSBoost classification results

Table 4 shows the micro‐averaged performance of K‐NN; again, *k*‐fold cross validation (*k* = 10) is employed. It can be noticed that K‐NN scored an overall accuracy of 83.5%, comparable to RF. Both models achieve very good performance on REM stage classification (F‐1 score > 0.75), with high precision and recall. As for RUSBoost, the dataset has been divided in 80% training and 20% test subsets. The epochs are randomly selected. The algorithm performance, summarized in Table 5, yielded an overall accuracy of 70.1%, impaired with respect to RF and K‐NN. This is not surprising, given the under‐sampling approach followed by this method and the small available data set. However, RUSBoost provides encouraging performance in terms of accuracy (89%), specificity (89.9%) and sensitivity (87.2%) on REM class, as can also be inferred by the confusion matrix reported in Figure 3. The REM stage is mistaken with *N*2 (3.7%), *N*1 (3.6%) and AWA (2.0%).A comparison between manual scoring and automated RUSBoost scoring is reported in Figure 4. It is worth noticing that the automatic scoring exhibits a more fluctuating trend if compared to manual annotation. This is quite reasonable, as manual annotation of hypnograms is driven by the human interpretation, which, for example, tends to rule out short AWA periods embedded in REM stages. In most papers addressing automatic hypnogram scoring, this phenomenon is by‐passed by adding a smoothing stage after the automatic scoring [33]. Finally, the performance yielded by our classifiers have been compared to those of already published methods. Study 1 [14] employs the same dataset as the present work (DREAMS Subjects Database), whereas Study 2 [12] was trained and tested on combined datasets of healthy controls (HC) and RBD patients (MASS, CAP and proprietary data collected at John Radcliffe Hospital, not publicly available). This dataset showed male predominance, while 80% of the participants in the DREAMS Subjects Database were female. Moreover, it employs features extracted from EEG, EOG and EMG signals; for all these reasons, only indirect comparisons can be done in this case.

**TABLE 3:** Performance of Random Forest classification

	**AWA**	**REM**	*N* **1**	*N* **2**	*N* **3**
**Accuracy**	0.977	0.927	0.940	0.869	0.954
**Sensitivity**	0.900	0.910	0.190	0.900	0.860
**Specificity**	0.980	0.950	0.960	0.840	0.976
**Precision**	0.880	0.760	0.540	0.840	0.900
**F1‐score**	0.890	0.828	0.281	0.869	0.880

**TABLE 4:** Performance of K‐NN classification

	**AWA**	**REM**	*N* **1**	*N* **2**	*N* **3**
**Accuracy**	0.979	0.925	0.943	0.871	0.953
**Sensitivity**	0.890	0.850	0.200	0.880	0.880
**Specificity**	0.989	0.940	0.990	0.861	0.971
**Precision**	0.910	0.740	0.570	0.850	0.880
**F1‐score**	0.900	0.791	0.296	0.865	0.880

**TABLE 5:** Performance of RUSBoost classification

	**AWA**	**REM**	*N* **1**	*N* **2**	*N* **3**
**Accuracy**	0.921	0.890	0.854	0.822	0.897
**Sensitivity**	0.498	0.872	0.436	0.579	0.984
**Specificity**	0.988	0.899	0.892	0.973	0.892
**Precision**	0.864	0.568	0.269	0.930	0.758
**F1‐score**	0.632	0.688	0.333	0.714	0.856

The results related to REM class are shown in Table 6. It can be noticed that all the three methods proposed in this paper outperform Study 1 [14], which is based on the same dataset, as for accuracy, sensitivity and specificity. Data regarding precision and F1‐score of Study 1 are not provided; nevertheless, RF and K‐NN exhibit reasonable precision and a good F1‐score. The performance of Study 2 refers to the HC group. It can be appreciated that, even though the proposed methods do not outperform [12] in overall accuracy of REM class, both RF and K‐NN exhibit higher sensitivity and comparable specificity and F1‐score. We deem these results quite remarkable, as our method is very simplified with respect to Study 2, and employs a single EEG channel for classification. Moreover, the combined dataset used in [12] encompasses 6360 observations for REM class, against only 1347 ones available in our data.

## 7 RESULTS ON RBD DATASET

The final objective of our work is to study pathological subjects, in particular those affected by RBD, with a single channel EEG. Hence, besides the preliminary test on the capability of our algorithms to correctly classify the five sleep stages in healthy subjects, we have considered the PSG of 22 subjects affected by RBD (19 males, aged 71 ± 6 years) enclosed in the CAP Sleep Dataset [34] available on PhysioNet [35]. EEG recordings related to the C3‐A2 channel (C4‐A1, if not available) were segmented into 30‐s epochs for feature extraction (cf. 4). The total number of available epochs was 14583; however, data exhibited a vast prevalence of the *N*2 class, with more than 5000 observations versus 688 of the least represented class, *N*1. As this could cause classification bias, our choice was to undersample the *N*2 observations to *N* = 2965, that is the mean number of observations in the other three classes. The total number of available epochs for feature extraction was 12277. The extracted features match those formerly implemented (cf. Table 2). Dimensionality reduction was performed, excluding NaNs and low‐variance features (cf. Section 4). Sleep stage classification is addressed by means of the three already introduced classifiers, trained on available data and validated through *k*‐fold cross‐validation, (*k* = 5). For the sake of brevity, Table 7 reports the results of the RF classifier only. The results of this model are rather satisfactory, with an overall accuracy of 87.11%.

**TABLE 6:** Performance comparison: RF, K‐NN, and RUSBoost (proposed) and already published methods

	**Study 1** ^*^	**Study 2**	**This study**		
**Classifier**	**Threshold**	**RF**	**RF**	**K‐NN**	**RUS**
**Accuracy**	0.885	0.96	0.927	0.925	0.89
**Sensitivity**	0.823	0.83	0.91	0.85	0.872
**Specificity**	0.893	0.98	0.95	0.94	0.899
**Precision**	N/A	0.84	0.76	0.74	0.568
**F‐1 score**	N/A	0.81	0.828	0.791	0.688

^*^ same dataset as our study

**TABLE 7:** Performance of RF classification on RBD subjects

	**AWA**	**REM**	*N* **1**	*N* **2**	*N* **3**
**Accuracy**	0.881	0.895	0.948	0.826	0.866
**Sensitivity**	0.852	0.581	0.411	0.650	0.761
**Specificity**	0.893	0.955	0.986	0.881	0.903
**Precision**	0.757	0.715	0.683	0.620	0.733
**F1‐score**	0.802	0.641	0.513	0.635	0.747

## 8 RESULTS OF BINARY CLASSIFICATION

**FIGURE 5:**
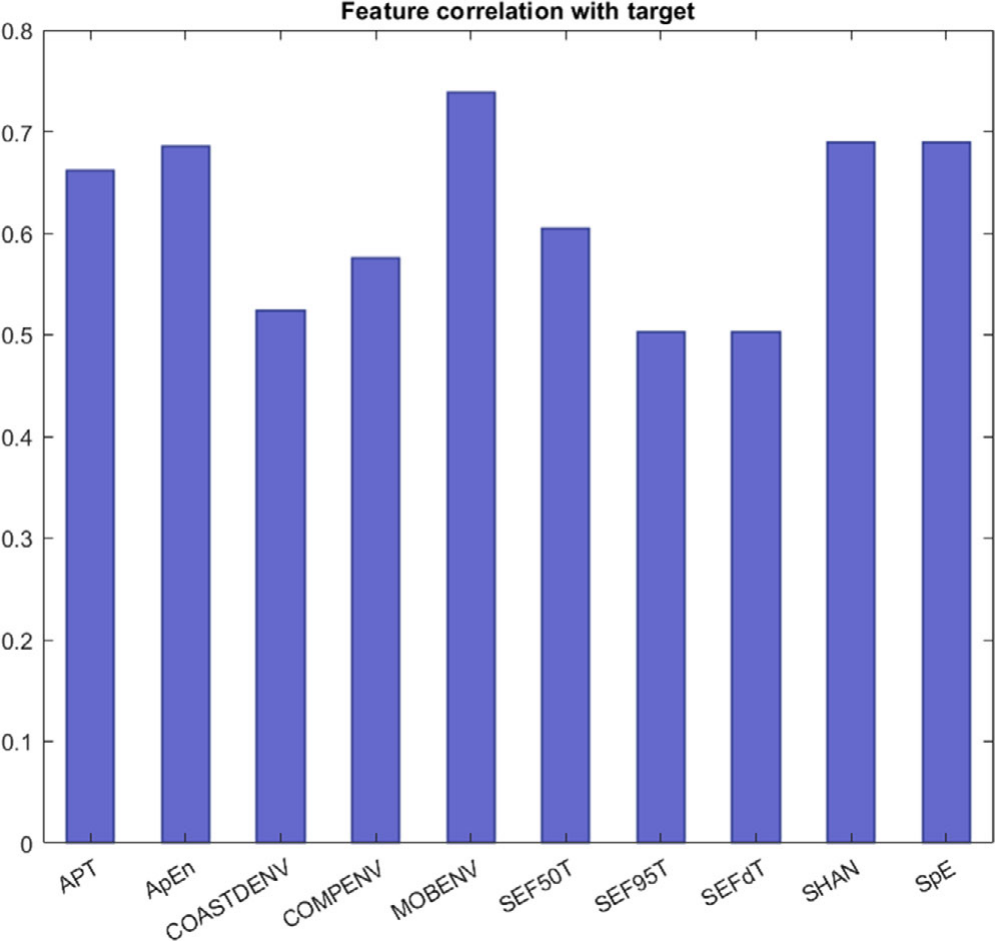
A set of novel features implemented and their correlation (Pearson) with REM class

As discussed in Section 3, we propose to exploit the dual nature of the REM stage (TREM and FREM micro‐structure) to enhance classification. To this end, a set of novel features describing the two micro‐states has been implemented in our model (cf. Table 2). To acknowledge the contribution of such features in the classification task, a binary classification problem has been set up, in order to distinguish between REM and NREM sleep. The wake stage has been discarded, and stages *N*1, *N*2 and *N*3 included in the NREM class. As we are dealing with a binary problem, we have tested K‐NN along with two further classifiers suitable for the problem at hand, namely Decision Tree (DT) and SVM. Each sample set was validated through Leave‐One‐Out cross‐validation (LOO‐CV), leaving out one observation at a time. The classification has been tested on both healthy and RBD subjects from the CAP Sleep Database. Results are displayed in Tables 8 and 9, in terms of accuracy, sensitivity, specificity, precision and mean squared error (MSE), that is test error. Sensitivity measures the performance on the REM class. It can be appreciated that REM detection efficiency is high in the control group, with DT yielding 97.9% sensitivity and the lowest mean squared error (cf. Table 8). Likewise, the performance on the RBD subjects is quite promising. Indeed, all three models yielded sensitivity in excess of 70%. The performance of K‐NN when the novel features are not implemented, are shown in Table 10. A slight yet measurable impairment in all the performance metrics can be appreciated in this case.Finally, feature correlation with target was computed, by means of Pearson correlation coefficient. Setting the target to REM class, all implemented features displayed good correlation. A set of these is displayed in Figure 5.

**TABLE 8:** Performance of binary classification on healthy controls from the CAP Sleep Database

	**Accuracy**	**Sens**	**Spec**	**Prec**	**MSE**
**K‐NN**	0,926	0,963	0,864	0,923	0,08
**DT**	0,949	0,979	0,897	0,941	0,05
**SVM**	0,863	0,935	0,741	0,859	0,13

**TABLE 9:** Performance of binary classification on RBD subjects from the CAP Sleep Database

	**Accuracy**	**Sens**	**Spec**	**Prec**	**MSE**
**K‐NN**	0,759	0,779	0,740	0,749	0,24
**DT**	0,765	0,767	0,763	0,764	0,23
**SVM**	0,643	0,702	0,582	0,627	0,36

**TABLE 10:** Performance of binary classification on RBD subjects from the CAP Sleep Database w/o implementation of novel features

	**Accuracy**	**Sens**	**Spec**	**Prec**	**MSE**
**K‐NN**	0,742	0,776	0,708	0,727	0,26

## 9 CONCLUSIONS

This work proposes an automatic sleep stage classification based on a single EEG channel. Three classification algorithms are addressed, namely RF, K‐NN and RUSBoost. Novel features are proposed, based on the micro‐structure of REM sleep. This represents a novelty, as REM sleep has always been considered a single, homogeneous stage. The achieved results reveal that all three methods achieve very good ability in detecting REM stage, with micro‐averaged accuracy of 92.7%, 92.5% and 89.9%, respectively. High sensitivity and specificity – with a satisfactory trade‐off between the two – underline good detection and a low number of false positives. This is demonstrated again (in RF and K‐NN) by the precision value, which is reasonable for experimental raw data (≈75%). The results outperform those published in [14] using the same dataset and are comparable with [12], where features from EEG, EOG and EMG signals are used. Finally, we have explored the capability of classifying REM versus NREM sleep stages, using data from both healthy controls and patients affected by RBD. The results are quite encouraging, and confirm the usefulness of the proposed features, based on fine REM sleep classification. In conclusion, the proposed methods is able to perform 5‐stage sleep classification in healthy controls using only one EEG channel; this a step forward towards the implementation of sleep measures at home, with a simplified sensor configuration. In fact, PSG is stressful for patients due to both the high number of electrodes and the diverse environment; it is costly, being performed in hospital and implying a time‐consuming manual annotation. Moreover, the performance on REM stage detection are promising in view of future studies on RBD in PD patients. Future developments will be in the direction of addressing a larger dataset, in order to validate the classification performance (in particular, those of the RUSBoost method, which suffers from data scarcity). Moreover, it is possible that the results are biased by the unbalanced sex ratio of the sample, as it was suggested that sleep is different in adults male and female subjects who do not have neurological disorders [36]; the verification of this point is left to future developments. Finally, we are setting up a protocol to train and validate our algorithm on PD subjects, using PSG signals as a term of comparison.
